# Whole-genome sequence of *Pseudomonas benzopyrenica* MLY92: isolation from diseased leaves of tobacco in China

**DOI:** 10.1128/mra.00176-24

**Published:** 2024-06-18

**Authors:** Juntao Gao, Yongfeng Jing, Zhijun Cheng, Ke Huang, Huilin Zhang, Yong Liu, Lei Yang, Shiwang Liu

**Affiliations:** 1 School of Biological & Chemical Engineering, Zhejiang University of Science & Technology, Hangzhou, China; 2 China Tobacco Hunan Industrial Co. Ltd, Changsha, China; The University of Arizona, Tucson, Arizona, USA

**Keywords:** *Pseudomonas benzopyrenica*, genome, gram-negative, tobacco

## Abstract

Here we present a sketch of the whole-genome sequence of *Pseudomonas benzopyrenica*. The strain comes from the leaf veins of a diseased tobacco plant. This study has significant research implications for gaining insights into the characteristics of microorganisms belonging to the genus *Pseudomonas*.

## ANNOUNCEMENT

*Pseudomonas benzopyrenica* belongs to the family *Pseudomonadaceae* in the phylum *Bacterial*, and has yellow, round, flattened colonies with smooth edges. The type strain BaP3 was isolated from soil samples in Hunan Province, China (1), and has been shown to effectively degrade benzo(a)pyrene (2). The strain described in this study was isolated from diseased tobacco leaves collected in Guizhou Province, China (25°02′24″ N, 104°54′00″E). The disease was initially characterized by the appearance of faded green water stains on the leaf veins, gradually expanding to form irregular necrotic spots on the margins of the leaf veins. Diseased leaf samples were sterilized with 75% ethanol, ground, and inoculated on NA agar (peptone 2 g, beef extract 0.6 g, sodium chloride 1 g, agar 3 g, distilled water 200 mL, pH = 7.0). Individual colonies were selected, and a single round flattened yellow colony was isolated and named MLY92.

The genomic DNA of MLY92 was extracted from an overnight culture in a liquid NA medium at 30°C using an MGIEasy Microbiome DNA Extraction Kit (MGI Tech, China). The DNA was sheared to 15–20 Kb using Megaruptor on the PacBio platform, size-selected, and accurately recovered with Sage ELF to produce HiFi libraries with highly uniform fragment sizes for sequencing. The raw sequencing data underwent screening to eliminate adaptor sequences and low-quality data fragments shorter than 1,000 bp, followed by the acquisition of SubReads (comprising 1,451,529 SubReads with an average length of 10,403 bp and an N50 value of 11,120 bp). These SubReads were subsequently self-corrected using Canu v1.5 and then assembled based on the self-corrected Corrected Reads. Corrected Reads were assembled (3–5), and gene prediction was conducted using Glimmer 3.02 (6–8). Annotation was conducted utilizing Diamond v0.8.31 in conjunction with database-specific characteristics (9–12), with the publicly accessible version annotated *via* PGAP (13). Average nucleotide identity (ANI) analysis was performed, and an ANI heatmap was generated using fastANI 1.32 with parameters set to -c 1024. TreeBeST 1.9.2 was employed to visualize the results obtained from Muscle 3.8.31 (parameters: treebest nj -b 1000, -in -out -maxiters 16). Multiple sequence comparisons were performed to generate statistical graphs depicting the number of homologous genes and phylogenetic tree diagrams. Genome circle diagrams were plotted using Circos 0.69–6. Default parameters were used unless otherwise specified.

Compared with publicly available genomes of closely related strains within *Pseudomonas oryzihabitans* (DE0585, DSM6835, FDAARGOS657, MS8) and *Pseudomonas rhizoryzae* (ZYY160) (14), ANI values ranged from 89.11% to 92.97%. Their gene family assignments showed a high degree of similarity. The genome sequence of MLY92 matched that of *Pseudomonas benzopyrenica* (BaP3) in GenBank with a 97.59% ANI value, indicating they are the same species.

**Fig 1:**
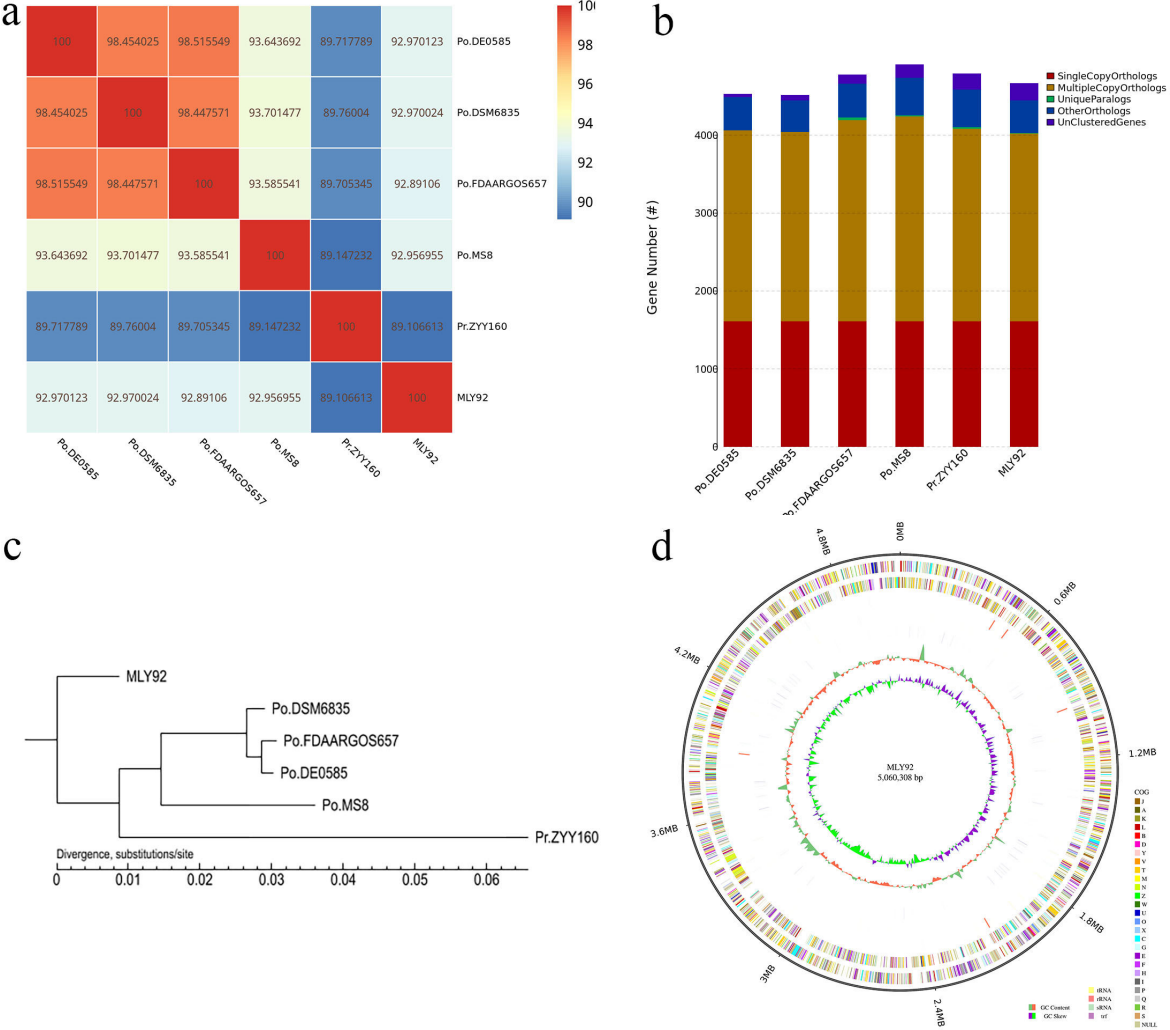
(a) ANI Heat map; (b) statistical graph of the number of homologous genes; (c) phylogenetic tree diagram; and (d) genome circle diagram.

The general genomic characteristics of MLY92 are shown in Table 1. Based on the sequenced bacterial genome sequence, GC skew analysis was performed using the (G-C)/(G + C) calculation method (15). Positive values suggest CDS transcription by the positive strand; negative values indicate the negative strand (Fig. 1d).

**TABLE 1:** Genome features of *Pseudomonas benzopyrenica* strains MLY92

Sample name	MLY92
Genome size (bp)	5,060,308
Coding sequences	4685
GC content (%)	65.5
Chromosome number	1
tRNAs	67
(5s,16s,23s) rRNAs	(5, 5, 5)
ncRNAs	7
Average length of 16S rRNA (bp)	1,525
Genome coverage	45.36
Number of pseudogenes	29

## Data Availability

The genome sequencing raw reads have been deposited in the NCBI Sequence Read Archive under accession numbers SRR26794288. The MLY92 genome sequence was deposited in the DDBJ/ENA/GenBank database under accession number CP145723 (BioProject/BioSample PRJNA1039312/SAMN38209873).
